# On‐Chip Thermoelectric Devices Based on Standard Silicon Processing

**DOI:** 10.1002/smll.202405411

**Published:** 2024-09-26

**Authors:** Elisabetta Dimaggio, Antonella Masci, Amedeo De Seta, Marc Salleras, Luis Fonseca, Giovanni Pennelli

**Affiliations:** ^1^ Dipartimento di Ingegneria della Informazione Università di Pisa Via G.Caruso I‐56122 Pisa Italy; ^2^ Instituto de Microelectrónica de Barcelona (IMB‐CNM, CSIC) Campus UAB Bellaterra Barcelona 08193 Spain

**Keywords:** power density, silicon nanobeams, seebeck voltage, thermal conductivity, thermoelectricity

## Abstract

The strong reduction of thermal conductivity with respect to bulk silicon makes nanostructured silicon one of the best materials for highly efficient direct conversion of heat into electrical power and vice‐versa. The widespread technologies for the integration of silicon devices can be used to define on‐chip micro thermoelectric generators (scavengers); similar structures could also be used for precise and well‐localized cooling through the reverse process of heat pumping. However, the road to the fabrication of integrated thermal energy scavengers or cooler, based on silicon, is still very long. In this work, the design and the fabrication process of on‐chip thermoelectric devices based on a large number of interconnected monocrystalline silicon nanobeams, very tall (>1 µm) and thin (less than 200 nanometers), arranged in large areas combs is shown. The small width of the nanobeams gives a reduced thermal conductivity, and the height perpendicular to the substrate allows the definition of a highly dense collection of nanostructures. The total cross‐section is far broader than that of other nanostructures, a characteristic that guarantees both mechanical stability and larger deliverable power per unit area.

## Graphical abstract

This experimental work focuses on the design and fabrication of a basic module for integrated thermoelectric devices based on a large number of interconnected monocrystalline silicon nanobeams, very tall (>1 µm) and thin (less than 200 nanometers). The proposed structure shows high mechanical stability and very good values of deliverable power per unit area.

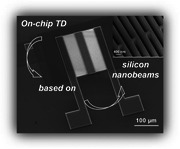

## 1 Introduction

The rapid development and commercialization of wireless devices for the Internet of Things should be accompanied by a strong advancement of energy‐scavenging solutions for their power supply.^[^ ^1^, ^2^ ^]^ Thermoelectric devices (TDs), which can convert heat directly into electrical power and vice versa, represent a great opportunity in this context, allowing electrical power production by exploiting every hot surface available, both in domestic and industrial environments.^[^ ^3^, ^4^ ^]^ To this end, it is fundamental to develop on‐chip integrated micro‐TDs that can be packed side‐by‐side with other integrated circuits for their autonomous, battery‐free supply.^[^ ^5^, ^6^ ^]^

Silicon, for its abundance and sustainability, is the basic material for integrated circuit fabrication, and the development of silicon‐based on‐chip devices can rely on a plethora of fabrication processes available for Nano and Micro Electromechanical Systems (NEMS/MEMS).^[^ ^7^ ^]^ Several solutions have been proposed for on‐chip thermoelectric microharvesters^[^ ^8^, ^9^, ^10^, ^11^ ^]^ based on a suspended active part bridging large silicon platform, used for the exchange of heat with hot and cold sources.^[^ ^12^, ^13^ ^]^ The fabrication follows the in‐plane strategy typical of the integrated circuits; to exploit this strategy, the heat must flow parallel to the substrate. This involves the suspension both of the active part and of, at least, one large silicon platform, to avoid the thermal conduction through the substrate. The heat paths need to be correctly designed in order to maintain high temperature differences between the ends of the active part, responsible for the thermal to electrical conversion. However, the presence of suspended parts, in particular of suspended large silicon platforms, results in very fragile and delicate devices.

Regarding the active part, thin films of conventional thermoelectric materials can be deposited by chemical vapor deposition or other techniques on suspended scaffolds of thermally insulating materials such as SiO_2_ or Si_3_N_4_.^[^ ^14^, ^15^ ^]^ However, standard materials are based on tellurium, which is rare, not environmentally friendly and poorly compatible with CMOS NEMS/MEMS technologies. Polysilicon,^[^ ^9^, ^16^, ^17^ ^]^ which has a reduced thermal conductivity with respect to bulk silicon for the scattering on the grain boundaries, can be used as an alternative. Indeed, polysilicon is a CMOS NEMS/MEMS compatible material. However, its electrical conductivity is also affected by the scattering on the boundaries, which has a deleterious effect on its thermoelectric performance. The winning strategy for on‐chip micro‐TDs is to directly use silicon also for the active part. When nanostructured, silicon is itself an excellent thermoelectric material,^[^ ^18^, ^19^, ^20^ ^]^ with a good figure of merit $ZT=S^{2}\frac{\sigma }{k_{t}}T$ (*S* Seebeck coefficient, σ electrical conductivity, *k*_*t*_ thermal conductivity, *T* absolute temperature). Nanostructured silicon retains the high electrical conductivity σ (when doped) of monocrystalline silicon, meanwhile the thermal conductivity *k*_*t*_ is strongly reduced due to the increase of the phonon scattering on the walls of the nanostructures. It is worth noticing that, in silicon, the phonon drag gives a non‐negligible contribution to the Seebeck coefficient (of the order of 20/30%).^[^ ^21^, ^22^ ^]^ Therefore, the decrease of the phonon propagation could reduce the Seebeck coefficient, but this effect is largely compensated by the strong reduction of the thermal conductivity.

Thermal conductivities smaller than 2 W (m K)^−1^ have been measured on nanowires.^[^ ^23^, ^24^ ^]^ Surface roughness has been proved to be very effective for thermal conductivity reduction.^[^ ^25^, ^26^ ^]^ Thermoelectric generators based on vertical silicon nanowires and nanostructures (heat flux perpendicular to the substrate) have been proposed.^[^ ^7^, ^21^, ^24^, ^25^ ^]^ Thin, rough silicon nanomembranes, in which heat transport occurs parallel to the substrate, showed *k*_*t*_ smaller than 20 W (m K)^−1^,^[^ ^27^ ^]^ Very thin suspended holey silicon nanomembranes,^[^ ^28^, ^29^, ^30^, ^31^ ^]^ with conductivities smaller than 10 W (m K)^−1^, can be fabricated on areas of several µm^2^ and arranged in parallel to the substrate.^[^ ^32^ ^]^ The reduced section of these membranes, which can be very large but must be thin to minimize the thermal conductivity, leads to a low electrical current, and, as a consequence, to a limited power output of these devices. Higher currents can be achieved by on‐chip TDs based on a large amount of silicon nanowires,^[^ ^33^, ^34^ ^]^ bridging large silicon platforms.^[^ ^35^, ^36^, ^37^ ^]^ The shortcomings of these solutions is the mechanical fragility of the overall device due to the suspension of the silicon nanostructures and of the large platforms needed for the heat exchange.

In this work, we present on‐chip TDs where the active part is based on a large number of monocrystalline silicon nanobeams. These nanobeams are fabricated in planar configuration (parallel to the surface): they are several tens of micrometers long (*L* = 50 µm) and have a very narrow (*W* < 200 nm) and tall (*th* > 1 µm) cross‐section. This cross‐section is close to that of the silicon nanostructures fabricated and analyzed in our previous work,^[^ ^27^ ^]^ in which the nanostructures had a cross‐section characterized by a width of about *W* = 1 µm and a thickness *th* between 100 and 200 nm. We demonstrated, there, the strong reduction of the thermal conductivity (well below 20 W (m K)^−1^), which is related with the small thickness *th* of the nanobeams, as shown by the theoretical considerations reported in the paper. We also reported experimental evidence of the effect of surface roughness, which increases the phonon scattering, decreasing the phonon conduction. In this work, we use structures(nanobeams) with very similar cross‐section, but in which *W* and *th* are inverted: the larger side is arranged perpendicular to the substrate, *th* = 1 µm, and the thinnest part is in plane, *W* < 200 nm. In this way, the reduced thermal conductivity, which is related to the width of the nanobeams, is preserved. On the other hand, the reduced *W* allows a high integration density: a large number of nanobeams can be placed very close to each other. Compared to strategies based on nanomembranes that are large and thin (see above), our strategy, based on narrow and tall nanobeams, allows a higher integration density, and, hence, a much larger total cross section on the same surface and a higher electrical current and power. For the fabrication, we exploit a top‐down approach: high‐resolution lithography is used for the narrow, planar face; highly selective plasma etching (Deep Reactive Ion Etching, DRIE) allows to define the tall vertical side. Suspended combs made of highly dense, parallel nanobeams can be easily fabricated on surfaces of several mm^2^. Large Si platforms are fabricated simultaneously with the nanostructure in a monolithic approach, so that there is crystalline continuity between the platforms and each nanobeam. The combination of high density and monolithic approach gives to the device a strong mechanical stability. Moreover, from an electrical perspective, the total cross‐section of the active area results very large. Therefore, the total current, and, hence, the delivered power for surface unit of this TD, is considerably high. Furthermore, the crystalline continuity between nanobeams and large platforms eliminates the electric losses due to the electrical contact resistance. Another key point of the device presented in this work is that the suspension of the large platform for the heat exchange is only partial. Even if the heath‐flow through the nanostructures is maximized when the platforms are completely suspended, we show that a partial suspension slightly affects the thermoelectric conversion efficiency, meanwhile it is crucial to enhance the mechanical stability of the device.

## 2 Device Concept

The device is fabricated following a top‐down approach based on standard NEMS‐MEMS fabrication techniques. High‐resolution lithography and highly selective deep‐reactive etching are used to define, in the top silicon layer of a Silicon‐On‐Insulator (SOI) substrate, a dense pack of nanobeams. These nanobeams are very long (several tens of micrometers) and have a very narrow and tall cross‐section. The thermal conductivity is strongly reduced due to the small width of the beams. The height of the cross‐section is determined by the thickness of the top SOI layer. The mechanical stability and a significant amount of electrical power output are achieved thanks to the height of the beams and to the high number of parallel structures packed together. These collection of nanobeams, which are the active part of the device, are fabricated to bridge large silicon platforms that allow the heat‐exchange with the sources.

**Figure 1:**
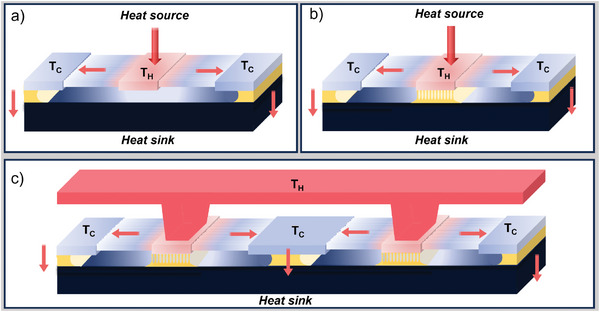
A sketch of the thermoelectric generator in a unileg configuration: a) fully suspended central silicon platform; b) partially suspended central silicon platform with a calibrated SiO_2_ underetch; c) multiple configuration of devices with a collector coupled with the heat source and aligned on the central silicon platforms.

The architecture of the device is depicted in **Figure** **1**a. The heat source is applied at the center of the structure on a suspended silicon platform that acts as a heat collector (T_*H*_). From the heated (T_*H*_) platform, the heat flows parallel to the substrate through two suspended combs of hundreds of nanobeams, reaches two lateral silicon platforms T_*C*_, and from there it flows perpendicularly to the substrate (silicon handle), which acts as heat sink. The lateral silicon platforms are anchored to the substrate through the buried silicon dioxide layer, and are made large enough to maximize heat dissipation. The best solution for the heat distribution would be to have a completely suspended central platform T_*H*_, so that all the heat flows through the nanobeams to the T_*C*_ platforms, parallel to the substrate, and from the *T*_*C*_ platform heat flows perpendicular to the substrate, as shown in Figure 1a. However, this solution gives problems of mechanical stability, resulting in a very delicate and fragile device. To improve the mechanical stability of this structure, crucial in particular when coupled with an external heat source, a partial suspension of the T_*H*_ platform can be performed through a calibrated under‐etching of the buried silicon dioxide. The resulting structure is shown in Figure 1b. The key‐point is to find the right trade‐off on the oxide etching, leaving enough oxide for a good mechanical consistency, but removing it as much as possible to limit the dissipation between the T_*H*_ platform and the substrate.

In this processing, we use a unileg configuration,^[^ ^37^ ^]^ with a single *n*‐type doping step. The Seebeck voltage generated by the application of a temperature difference can be measured between T_*H*_ and each T_*C*_, where metal contacts are fabricated. In principle, as shown in Figure 1c, several legs connected electrically in series and thermally in parallel can be fabricated on the same chip, in order to increase the Seebeck voltage. It is straightforward to dope the legs either *n* or *p* type, in alternate configuration, exploiting conventional selective doping techniques. The legs can be coupled with an external heat source through a heat collector that can be fabricated either in metal or in silicon, exploiting basic MEMS processes. The silicon collector can be connected to the T_*H*_ platform by flip‐chip bonding. It is important to highlight that the device exploits in full the thermal potentialities of silicon: the reduced thermal conductivity of the very thin nanobeams, which are the active part of the device, makes possible to establish a high‐temperature difference between their ends. On the other hand, the macroscopic silicon platforms and heat collectors retain the high thermal conductivity of bulk silicon, which is optimal to achieve a good and uniform temperature distribution for a better heat exchange.

### 2.1 Fabrication Steps

**Figure 2:**
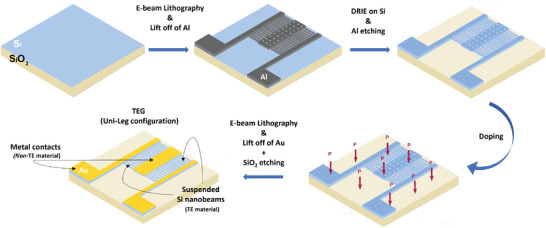
Sketches of the fabrication steps: starting from the definition of the nanobeams through e‐beam lithography and DRIE, followed by phosphorous doping and ending with the metal electrode definition and suspension of the entire TD.

**Figure 3:**
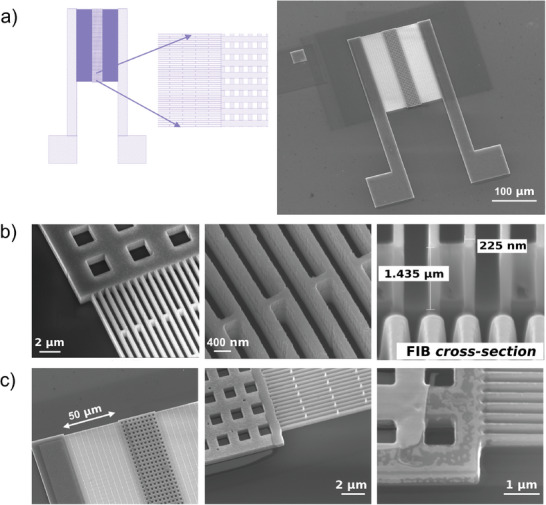
Images of the device throughout the fabrication process: a) layout of the first high resolution layer, which shows the structure of the TD and a SEM picture of the final device; b) details of the nanobeams after the dry etching step at different magnification and a FIB cross section of the beams; c) details of the structure after the metal contact definition and the etching of the buried oxide for the suspension.

A sketch of the process sequence is shown in **Figure** **2**. The device has been fabricated on a SOI wafer, with a 1µm thick top silicon layer, a 2 µm thick buried oxide layer and a handle substrate 500 µm thick, cut in chips of roughly 1 cm^2^. The width of the nanobeam cross‐section is determined in the e‐beam lithographic step. The smaller, the lower thermal conductivity can be expected. The height of the nanobeam cross‐section is determined by the thickness of the top silicon layer. The higher, the better, because a higher electrical current (higher electrical power) can be delivered, and a higher heat flux can be handled. It must be mentioned that the ratio between the electrical and thermal conductances, which determines the conversion efficiency, is the same than the ratio between the electrical and thermal conductivities. Aluminum strips narrower than 200 nm, 50 nm thick, and 50 µm long have been fabricated by high‐resolution e‐beam lithography and lift‐off in hot acetone, and used as a mask for the deep reactive ion etching (DRIE). The geometry that has been defined is shown in the second step of Figure 2: two aluminum arrays of strips are placed between a common holey body, which is defined as the central T_*H*_ platform, and two lateral collectors (T_*C*_ platforms), which end on larger areas where the electric pads are defined in the last fabrication steps. A DRIE process has been used to transfer the geometry of the aluminum film to the top silicon layer: the width and length of the nanostructures are related to the previous lithographic step, the height can reach at most the top silicon layer thickness of 1 µm. After the DRIE, the aluminum mask has been removed by wet chemical etching. As a result, the geometry is transferred on the silicon top layer, as visible in the third step of Figure 2 and in the SEM images of **Figure** **3**a,b. In the picture on the left of Figure 3a the layout of the first lithographic step is reported. In Figure 3b a FIB cross‐section of the beams is also reported. The strong thermal conductivity reduction relies on the width of the aluminum nanostrips, which determines the width of the etched silicon nanobeams. The holes through the central platform are fundamental for the final suspension of the structure, as they allow the partial buried oxide underetching. The width and, in particular, the pitch of the holes has been designed big enough to maintain the high thermal conductivity of silicon for a good heat redistribution/exchange. The fourth step of Figure 2 shows the device doping with a pre‐deposition step at 700°C for 10 min from a phosphorous solid ceramic source, followed by a drive‐in step consisting in a Rapid Thermal Oxidation (RTO) at 1150°C. The oxide grown during this RTO, roughly 30 nm thick, prevents the out‐diffusion, further reducing the width of the nanobeams (roughly 30 nm), and it is removed before metal contact definition. The *n* doping concentration resulted in 10^19^ cm^−3^. At the end, metal contacts have been fabricated by e‐beam lithography, metal evaporation (10 nm of Cr for adhesion plus 80 nm of Au) and lift‐off, as shown in the final step of Figure 2. Finally, a buffered hydrofluoric acid (BHF) etching has been performed for the removal of the buried oxide. The etching time has been accurately calibrated to allow the complete suspension of the nanobeams, but to have a suitable oxide left under the central T_*H*_ silicon platform that results only partially suspended at the end of the processing. Etching times are reported in the result section. Further details on the fabrication process can be found in the Experimental Section. It is worth mentioning that the nanobeams are much narrower than the distance between the windows through the central silicon platform, hence the oxide of the large arrays of nanostructures is removed in a minimal etching time. In Figure 3c SEM images of a typical final device are reported, where details of the holey central structure and of the BHF etching for suspension are shown.

### 2.2 Finite Element Simulations

**Figure 4:**
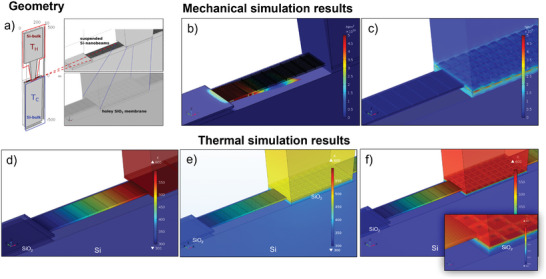
a) The overall simulated geometry, which represents the device coupled with silicon heat‐exchangers. The enlargement shows the top silicon exchanger T_*H*_ positioned on the device central T_*H*_ platform. The T_*C*_ exchanger is the substrate (silicon handle). b,c) Mechanical simulation results, with a pressure of 1 MPa applied on the upper surface of the top silicon exchanger. In Panel (b) the silicon dioxide has been completely removed under the central T_*H*_ on‐chip platform (fully suspended), and the structure collapses. In Panel (c) a portion of silicon dioxide is left (partially suspended). Panel (d), (e), and (f) show thermal simulations, with T_*HOT*_=600 K (on the top of the hot silicon block) and T_*COLD*_=300 K (on the bottom of the silicon handle). In Panel (d) there is no silicon dioxide underneath the central silicon platform, and this maximizes the temperature drop between the ends of the nanobeams; in Panel (e) the silicon dioxide is completely left underneath the central silicon platform, and this strongly penalises the temperature drop, which is smaller than the T_*H*_‐T_*C*_ difference of the source; in Panel (f) the silicon dioxide has been only partially removed (through the holey central silicon body). The simulation result in (f) shows a good compromise between mechanical strength and temperature drop between the nanobeams, which is only slightly affected by the oxide left under the T_*H*_ platform.

The device has been designed through Finite Element Simulations (FEM) with COMSOL MULTIPHYSICS, in order to verify the good mechanical stability and optimize the heat fluxes. In panel a) of **Figure** **4**, the complete simulated geometry is shown. Three main parts can be identified: the TD core, defined on a SOI substrate, and two blocks of bulk silicon, acting as heat exchangers: they allow the coupling of the TD with the hot source and the cold sink. The T_*H*_ block is positioned on the central T_*H*_ platform, as shown in the enlargement of panel (a). The T_*C*_ block is the silicon substrate (handle layer of the SOI wafer). This is only a small portion of the final device: in order to limit the numerical weight of the simulation, only 100 parallel nanobeams are considered. The simulation is anyway significant to represent devices with a higher number of parallel nanobeams, where mechanical and thermal phenomena are only scaled. In the enlargement of panel (a), it is shown one of the two suspended combs of 100 nanobeams, each of 50 nm width, 50 µm length (between the platforms) and 1 µm deep, connected together to the central T_*H*_ silicon platform (30 µm wide and 500 µm long) on the right and to the lateral silicon platform T_*C*_ on the left. A layer of silicon dioxide, with dimensions 10 µm x 100 µm x 2 µm, has been placed between the two lateral collectors and the Si‐substrate heat sink: this layer represents the leftover buried oxide of the SOI wafer after the BHF etching. Two aspects of the device have been refined and optimized through FEM simulations: 1) mechanical stability and 2) maximization of the temperature difference between the ends of the nanobeams. This temperature difference should be as close as possible to the temperature drop between the hot source and the cold heat sink. Mechanical simulations, with the COMSOL solid mechanics model, have been carried out in order to evaluate the mechanical displacement of the nanostructures under a normal Pressure of 1 MPa, applied on the upper surface of the top silicon T_*H*_ block; this is a typical pressure value considered for the commercial TDs, based on conventional thermoelectric materials. Figure 4b,c shows a comparison between the simulation performed on a device with a completely suspended T_*H*_ platform and a device in which some silicon dioxide has been left under this central platform. Nanobeams are always completely suspended, bridging the T_*H*_ and T_*C*_ platforms. The comparison gives evidence that the oxide layer prevents the collapse of the nanostructures and increases the mechanical stability of the overall device. In particular, with no oxide left under the central T_*H*_ platform, the device collapses.

The temperature distribution has been determined by the COMSOL temperature model, with the constraints of a hot temperature of 600 K applied on the top silicon block and a cold temperature of 300 K fixed on the bottom silicon block. Hence, the simulation takes into account the complete heat flux, from the source where the top silicon block is applied to the sink under the silicon handle substrate. The thermal conductivity k_*t*_ of the bulk silicon has been fixed at 148 W (m K)^−1^, while for the nanobeams a k_*t*_ value of 20 W (m K)^−1^ has been considered, which is in line with experimental results achieved on rough silicon nanomembranes.^[^ ^38^ ^]^ Indeed, this is a rather prudential value because smaller *k*_*t*_ have been measured in nanomembranes and, in particular, in rough silicon nanowires. To ensure that the leftover silicon dioxide does not cause extra heat losses through the substrate, a comparison of the thermal distribution along the combs in three different cases has been performed:

1. Figure 4d: total suspension. The silicon dioxide is completely removed under the central T_*H*_ platform, which is fully suspended. This is the best situation for the temperature distribution, but it is non convenient for the reduced mechanical stability (see Figure 4b).
2. Figure 4e: no suspension. The silicon dioxide is completely left under the central body. This gives an excellent mechanical stability, but part of the available temperature difference between the sources is wasted for the heat conduction through the oxide layer under the T_*H*_ platform toward the substrate.
3. Figure 4f: partial suspension. The silicon dioxide is partially etched through the holey central silicon platform; the mechanical stability is good, and only a small amount of heat flux is wasted passing directly from the T_*H*_ platform to the substrate. The amount of the oxide left under the central platform has been found to be the best trade‐off between the optimization of the heat flux and of good mechanical strength. The inset of Figure 4f shows that the greatest part of the original T_*H*_ ‐T_*C*_ temperature falls across the nanobeams, as expected; hence, the voltage output (Seebeck voltage) is maximized and almost proportional to the original T_*H*_ ‐T_*C*_ difference between the source and the sink.

As a result of these simulations, the partial presence of silicon dioxide, which improves the mechanical stability, does not affect the functionality of the device and can be kept during the fabrication process.

## 3 Measurements and Results

**Figure 5:**
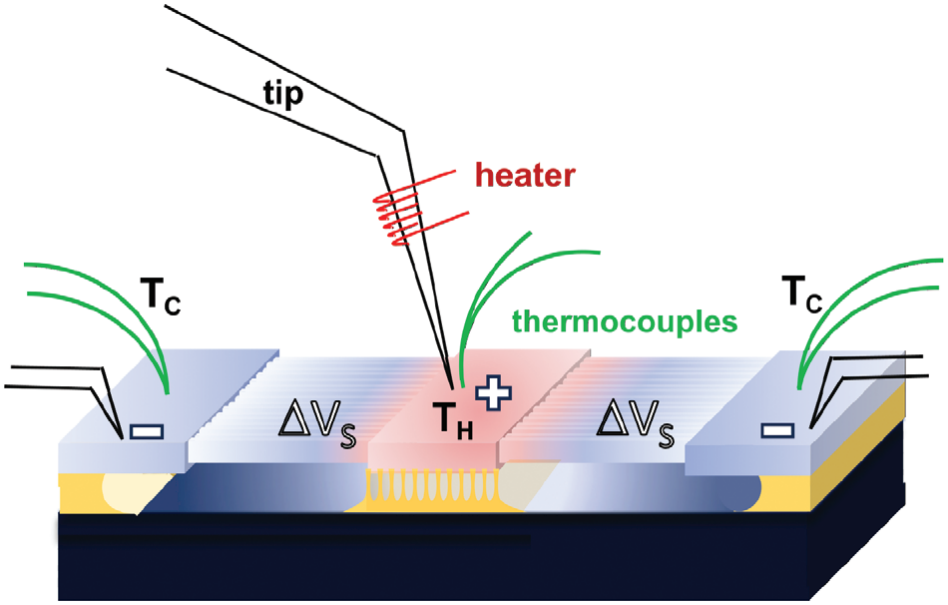
Sketch of the device showing the measurement setup.

Seebeck measurements have been performed on the suspended devices shown in Figure 4, characterized by the two combs of nanobeams, each covering an area of 200 µm × 50 µm, with 100 nanobeams per comb, connected together by T_*H*_ platform (200 µm × 30 µm) and ended on the opposite sides with T_*C*_ contacts of 350 × 30 µm^2^ and two 100 µm^2^ pads. The measurement set‐up is represented in the sketch of **Figure** **5**. Micromanipulators, with a tip radius of about 1 µm, have been used for the measurement of both the temperatures T_*H*_, through standard macroscopic K‐thermocouples, and of the Seebeck voltage Δ*V*_*S*_. The tip positioned in the central, almost‐suspended, *T*_*H*_ platform is heated, and so it is also used to inject heat into the device. Heat flows through the nanobeams, parallel to the substrate, from the central platform to the two side platforms anchored to the substrate through the SiO_2_ layer. From these side platforms the heat is dissipated perpendicularly to the surface, toward the substrate as explained in the Device Concept section. The substrate, acting as heat collector, is mounted on a heat sink maintained at a constant low temperature *T*_*C*_ = 20°C.

**Figure 6:**
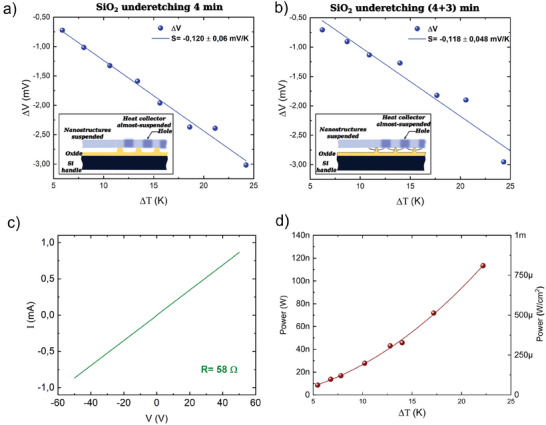
Measurement results. Voltage drop as a function of temperature drop between the central platform T_*H*_ and one of the two lateral T_*C*_ platforms, in case of SiO_2_ under etching performed for 4 min(a) or 7 min(b). The slope of the linear fit gives an estimation of the Seebeck coefficient in the two cases. c) The *I–V* curve of a typical device, measured at ΔT = 0. d) The Output power, on the left‐y axis, and the output power density, on the right‐x axis, as a function of temperature drop. The fitting curve shows the dependence of the output power on the ΔT^2^.

We measured the voltage drop between the T_*H*_ platform and one of the two lateral T_*C*_ platforms, for several temperature differences. No significant changes have been noted in choosing the left or the right T_*C*_ platform. Typical values of voltage drop, as a function of temperature differences, are reported in the graphs of **Figure** **6**a,b. The Seebeck coefficient is determined by a linear fit of these graphs (see the legend). We assume that: 1) the temperature of the large area platforms is uniform, because they are quite thick (1 µm) and, hence, silicon retains its good thermal conductivity. Moreover, they are covered by a conductive gold layer 80 nm thick, so that a potential residual small temperature gradient would not affect the electric potential, because the Seebeck coefficient of metals is very small (order of µV K^−1^). 2) As a consequence of the first assumption, the Seebeck voltage drop is located between the ends of the nanostructures, which conversely have a very high‐temperature gradient, as demonstrated by finite‐element simulations, due to their very low thermal conductivity. Therefore, the micromanipulated tips measure the effective voltage drop between the ends of the nanostructures. The measured voltage drop is proportional to the temperature difference between the ends of the nanobeams (internal temperature drop), which is smaller than the externally imposed temperature difference *T*_*H*_ *‐T*_*C*_. The difference between the external and internal temperature drops is due to losses on the central and lateral platforms. As demonstrated by FEM analysis, these losses are small because the platforms have high thermal conductivity. Note that only the heat flux flowing through the nanobeams (parallel to the substrate) is useful for thermal to electrical (or vice versa) energy conversion. Part of the heat generated by the hot source is wasted because it flows perpendicularly from the central platform to the substrate through the remained oxide. To verify that this wasted heat does not have a significant effect on the device performance, we performed the following test. A first SiO_2_ under‐etching (through BHF) has been performed before the measurement. The under etching time (4 min) is long enough to suspend the nanobeams, which are 150 nm wide, but it is not sufficient to completely remove the oxide under the central T_*H*_ platform that remains sustained by oxide pillars left between the windows. This is because the width and distance of the windows through silicon on the T_*H*_ platform (see SEM photos of Figure 3) have been optimized in the finite‐element simulations: they are wide enough to allow the penetration of BHF for the oxide under etching; their distance is designed to be large enough to not significantly reduce the high thermal conductivity of the large silicon T_*H*_ platform. As demonstrated by FEM simulations, the remained oxide with 4 min underetching does not significantly affect the heat flux through the nanobeams. In order to confirm this vision, after the first measurement, a second BHF underetching has been performed in order to further reduce the oxide under the platform, but still not completely. A second measurement run has then been applied, and results are shown in Figure 6b. By imposing the same external temperature drop, the same Seebeck coefficient (Seebeck voltage) was achieved, within the experimental deviation. Therefore, the internal temperature difference between the ends of the nanobeams is the same and is not affected (or only slightly affected) by the leftover oxide. Hence, the main heat path flows through the suspended nanostructures, while the wasted heat through the residual oxide under the central platform does not heavily affect the performance of the device. In order to evaluate the capabilities of the device in terms of the output power, at first we measured the *I* − *V* characteristic at uniform temperature, shown in Figure 6c for a typical device. As expected, for heavily doped silicon, the characteristic is linear, and its fit gives the thermoelectric generator resistance, which is 58 Ω for the device of Figure 6. Considering the geometrical factors (number of parallel nanobeams, length and cross‐section surface), this resistance gives an electrical resistivity of 1.7 × 10^−3^ Ωcm (conductivity σ of the order of 600 Ω^−1^cm^−1^). This resistance is consistent with a doping concentration of about 5 × 10^19^ cm^−3^, compatible with the performed doping process. The large area platforms are covered by a metal film, hence their electrical resistance is very low. Figure 6d reports the output power of a typical device, as a function of the temperature difference between the central platform and the lateral contacts. The left‐y axis reports the device output power. The right‐y axis reports the output power normalized with respect to the device area. This output power density as a function of the temperature difference, is measured through macroscopic thermocouples applied to the tips. For this calculation an area of 80x200 µm^2^ has been estimated, which takes into account a single comb plus half of the T_*H*_ and T_*C*_ platforms on its sides. The power density shows the expected quadratic dependence of the output power on the temperature difference, with a specific generated power of 4.5 µW cm^−2^ K^−^2.

## 4 Conclusion

We have presented the design, fabrication process and characterization of an all‐silicon micro‐thermoelectric generator, integrated on‐chip through advanced micro and nano fabrication techniques. The main advantage of this device is that silicon is both the structural and functional material: it does not require any traditional thermoelectric material, such as tellurium or lead compounds, which are not compatible with on‐chip integration and moreover arise issues of availability, toxicity and sustainability. The thermal properties of silicon are fully exploited. On the same chip, large silicon platform with high thermal conductivity are used where an uniform temperature distribution is required; nanostructured silicon beams are used where a reduced thermal conductivity is required to achieve high temperature differences, in order to generate high Seebeck voltage output. In general, this dual high/low thermal conductivity of silicon can be used for the design of on‐chip devices with optimized heat paths, for an optimal exploitation of heat sources. The measured generated electrical power reaches values close to 1 mW cm^−2^, with available temperature differences on the order of a few tens of degrees: these temperatures are well within the range available in many industrial and/or domestic environments. This generated power is enough to supply small systems, such as sensor nodes or similar. Hence, on‐chip micro‐TDs can be used side‐by‐side with other chips for their supply through scavenging from a hot surface. TDs based on the same fabrication principles can also be used for heat pumping, exploiting the dual phenomenon of thermoelectric generation, that is thermoelectric cooling. A supplied TD can be used for localized and well controlled cooling of other chips (integrated circuit). Almost‐suspended silicon platforms can be flip‐chip bonded directly on the chip to be cooled, and more precisely on the areas of the chip where heat is mainly generated, for a micro‐localized cooling.

## 5 Experimental Section

### Nanobeams Definition

Two combs of nanobeams were defined applying a highly selective etching on the silicon top layer of a SOI wafer, through an aluminum mask defined by electron‐beam lithography, aluminum evaporation and lift‐off. Lithography was implemented with standard double layer PMMA resist (996k MW 3% in anisole, on 350k MW 3% in anisole), developed in 3:1 IPA:MIBK. Exposure was performed with a SEM‐FEG JEOL 6500F equipped with a pattern generator. To be noted that the as the nanostructure width is in the order of 200 nm, advanced optical lithography can be used as alternative for mass production. A tailored DRIE process, for 2:45 min, was performed using the buried oxide as an etch‐stop layer.

### Aluminum Etching

After the DRIE, the aluminum mask was removed with a wet chemical etching (80:5:5:10 H_3_PO_4_:HNO_3_:HAc:H_2_O) performed at ^43○^C for 5 min, alternated with a rinse in DI water every 30 s to reduce the effect of the hydrogen bubbles in the etching uniformity. Finally, the chips were cleaned in acetone and IPA, and blow‐dried with pure nitrogen.

### Metal Contact Definition

A final low‐resolution lithographic step was used to define the metal contacts on the two pad areas and on the central silicon body that connects the two nanobeam combs. After the resist development in 1:1 IPA:MIBK, a layer of 10 nm of Cr (for adhesion) and 80 nm of Au was thermally evaporated on the entire surface, followed by a lift‐off step in hot acetone.

### Electrical and Thermal Measurements

Thermal measurements were performed with K‐type thermocouples applied on the micromanipulated tips. The temperature was measured by a Siglent SMD 3065. Heating was applied to the central silicon platform, supplying current to a constantan wire wrapped around the tip directly in contact with it. A remotely electrical power supply was used to regulate the wire heating. This technique is not suitable for thermoelectric efficiency measurements, since most of the heating is dissipated through the tip and manipulator. Seebeck voltage measurements were performed through a high precision Nanovoltmeter (Keihtley 2182). *I–V* characteristics have been measured through a source measurement unit (Keihtley 2602).

## Conflict of Interest

The authors declare no conflict of interest.

## Data Availability

The data that support the findings of this study are available from the corresponding author upon reasonable request.
